# Transcriptome analysis of sheep oral mucosa response to Orf virus infection

**DOI:** 10.1371/journal.pone.0186681

**Published:** 2017-10-26

**Authors:** Huaijie Jia, Leilei Zhan, Xiaoxia Wang, Xiaobing He, Guohua Chen, Yu Zhang, Yuan Feng, Yaxun Wei, Yi Zhang, Zhizhong Jing

**Affiliations:** 1 State Key Laboratory of Veterinary Etiological Biology, Key Laboratory of Veterinary Public Health of Ministry of Agriculture, Lanzhou Veterinary Research Institute, Chinese Academy of Agricultural Sciences, Lanzhou, Gansu, China; 2 Center for Genome Analysis, ABLife Inc., Wuhan, Hubei, China; 3 School of Public Health, Faculty of Medicine, Lanzhou University, Lanzhou, Gansu, China; 4 Laboratory for Genome Regulation and Human Health, ABLife Inc., Wuhan, Hubei, China; CEA, FRANCE

## Abstract

Contagious ecthyma is a highly contagious disease with worldwide distribution, which is caused by the Orf virus (ORFV) belonging to the *Parapoxvirus*. To study the alteration of host gene expression in response to ORFV infection at the transcriptional level, several young small-tailed Han sheep were inoculated with ORFV, and their oral mucosa tissue samples (T0, T3, T7 and T15) were collected on day 0, 3, 7 and 15 after ORFV infection respectively. RNA-seq transcriptome comparisons were performed, showing that 1928, 3219 and 2646 differentially expressed genes (DEGs) were identified among T3 vs. T0, T7 vs. T0, and T15 vs. T0 respectively. Gene Ontology (GO) analyses of the DEGs from these comparisons, revealed that ORFV might provoke vigorous immune response of the host cells during the early stage of infection. Moreover, GO and network analysis showed that positive and negative regulative mechanisms of apoptosis were integrated in the host cells through up or down-regulating the expression level of DEGs involved in apoptotic pathways, in order to reach a homeostasis of oral mucosa tissues during the exposure to ORFV infection. In conclusion, our study for the first time describes the direct effects of ORFV on the global host gene expression of its host using high-throughput RNA sequencing, which provides a resource for future characterizing the interaction mechanism between the mammalian host and ORFV.

## Introduction

Contagious ecthyma also known as Orf, is a non-systemic cutaneous and debilitating disease with worldwide prevalence, causing serious financial losses in livestock production. It is a zoonotic disease which mainly infects goats and sheep, but other various ruminants and mammals, like camel, deer, reindeer, muskox, serow, dog, cats and squirrel, have also been reported to be infected [1,2]. Moreover, people who come in direct or indirect contact with infected animals have the possibilities to be infected [3]. Clinically, the lesions of the Orf tend to initially proliferate on the mouth and oral mucosa as well as around the nostrils followed by the formation of papules, vesicles, pustules with a yellowish creamy appearance and scabs that finally become dry and with no scar remaining [2], and the development pattern occurs in a period of one or two months.

Previous studies have revealed that Orf was caused by the epitheliotropic Orf virus (ORFV) [4,5], a species of *Parapoxvirus* from Poxviridae. The ORFV has a double-stranded DNA genome of approximate 140 kilo-base pairs, encoding 132 genes. ORFV infection mostly provokes a vigorous skin immune response of the host. However, the ORFV can limit the effectiveness of host immunity and allow time for virus replication through some molecular mechanisms [5,6]. Chemokines activate and mediate inflammation induced leukocyte recruitment to infectious sites as well as homeostatic migration of leukocytes through lymphoid organs [7], but the ORFV produces a soluble secreted chemokine binding protein (CBP) which is capable of disrupting chemokine gradients thus blocking the trafficking of immune cells to infectious sites [8]. It has been discovered that *ORFV020* has a role in interferon resistance by inhibiting the activation of IFN-inducible dsRNA-dependent kinase in sheep [4]. NF-κB regulates the expression of an impressive range of cellular genes which are of great significance for early anti-viral and inflammatory responses, and previous studies revealed that ORFV encoded three inhibitors of NF-κB signaling pathway [9–11].

Apoptosis is a rather important process in multicellular organisms, and it can remove the old, unwanted, or potentially dangerous cells. It plays a crucial role in the development and homeostasis of tissues, as well as in immune responses to pathological signals [12,13]. Apoptosis is of great importance in host defenses against virus infection through executing the cell suicide program in order to block virus replication in infected cells [14]. Therefore, it is reasonable that viruses have evolved multiple regulators that are able to block apoptosis at different stages within the apoptotic pathways [14,15]. Primary Orf infections typically need two months to resolve, but chronic and persistent infections have also been recorded [5], which implied that there might be some molecular mechanisms of anti-apoptosis in ORFV-infected cells. Interestingly, it was found that the ORFV can produce an inhibitor of apoptosis, and ORFV-infected cells were completely resistant to the UV-induced changes in cell morphology, caspase activation, and DNA fragmentation [16–18].

With the rapid development of high-throughput RNA sequencing (RNA-seq) technology, we now are able to explore the comprehensive transcriptional landscape in the host upon virus infection [19–21]. In the current study, RNA-seq performed on the oral mucosa tissues of ORFV-infected sheep was applied to investigate the systemic alteration of the host gene expression. These data are available to explore the interaction mechanism between the host and ORFV, and provide valuable information for its treatment and prevention.

## Materials and methods

### Ethics statement

Sheep were sampled with signed consent from animal breeding center of Lanzhou Veterinary Research Institute under an approved protocol of PR China for the Biological Studies Animal Care and Use Committee.

### Experimental animals and virus strain

6 male small-tailed Han sheep, 4 months old, were purchased from the animal breeding center of Lanzhou Veterinary Research Institute. No ORF disease had been previously observed in these sheep. The body temperature, mental status and feed intake of each sheep were monitored twice a day (at 9 a.m. and 3 p.m.) until the end of experiment.

To ameliorate the suffering of these sheep, we have made them acclimatized for two days before the initiation of the study in the animal biosafety level 3(ABSL-3) laboratory with standard laboratory chow and water, ad libitum. ORFV strain (ORFV/QH01/2010) was isolated from the scar of a clinically ORFV infected sheep from Qinghai, China, and cultured in Hela cells.

### Experimental inoculation of sheep with ORFV

Prior to infection, 6 sheep were injected with Lumianning (an anesthetic drug) via intramuscular injection. After narcosis, each sheep was inoculated with 0.2×10^5.5^ TCID_50_ ORFV via the oral mucosal scratch, which were gently pre-made by disposable syringe needle. During the infection period, typical cutaneous lesions were observed only in oral mucosa. No systemic symptom, like high fever, was observed. None of the animals died prior to the experimental endpoint.

0, 3, 7 and 15 days after ORFV infection, the oral mucosa tissue (50 mg per sheep) from each infected sheep was collected under narcosis condition. Tissues from three sheep were pooled as one RNA-seq sample for each time point.

### RNA extraction and sequencing

The tissue samples were ground (with mortar and pestle, under continuous liquid N_2_ chilling) into fine powder before RNA extraction. Total RNA was extracted from 30 mg of the ground tissue by using hot phenol method. The RNA was further purified with two phenol-chloroform treatments and then treated with RQ1DNase (Promega, Madison, WI, USA) to remove DNA. The quality and quantity of the purified RNA were redetermined by measuring the absorbance at 260 nm/280 nm (A260/A280) using Smartspec Plus (BioRad, USA). The integrity of RNA was further verified by 1.5% agarose gel electrophoresis.

For each sample, 10μg of the total RNA was used for RNA-seq library preparation. Polyadenylated mRNAs were purified and concentrated with oligo(dT)-conjugated magnetic beads (Invitrogen, Carlsbad, CA, USA) before directional RNA-seq library preparation. The purified mRNAs were then iron fragmented at 95°C followed by end repair and 5' adaptor ligation. Then, reverse transcription was performed with RT primer harboring 3' adaptor sequence and randomized hexamer. The cDNAs were purified, amplified, and stored at -80°C until they were used for sequencing.

For high-throughput sequencing, the libraries were prepared following the manufacturer's instructions. Illumina Nextseq 500 system was used to collect data from 151-bp pair-end sequencing (ABlife Inc., Wuhan, China).

### Bioinformatic analysis

RPKM (reads per kilobase per million mapped reads), was used to evaluate the expression level of genes. To measure the RPKM value and screen out the differentially expressed genes (DEGs), we applied the software edgeR[22], which is specifically used to analyze the differential expression of genes using RNA-Seq data. The genes with RPKM< 0.1 in every sample were removed before analysis. To determine whether a gene was differentially expressed, we analyzed the results based on the fold change (fold change ≥2 or ≤0.5) and *P*-value (*P***≤**0.01).

To predict the gene function and calculate the functional category distribution frequency, Gene Ontology (GO) analyses were employed using DAVID bioinformatics resources [23]. The networks were constructed by calculating the Pearson correlation coefficient (PCC) of the DEGs. Cytoscape was used to display the co-expression network [24].

### Validation of DEGs by qRT-PCR

In this study, to elucidate the validity of the RNA-seq data, quantitative real-time PCR (qPCR) was performed for some selected DEGs, and normalized with the reference gene *GAPDH* gene of sheep. The information of primers is presented in Table 1. The same RNA samples for RNA-seq were used for qPCR. In each pooled sample, l μg of RNA was reversely transcribed using the PrimeScript^TM^ RT Reagent Kit (Takara, Dalian, China) following the manufacturer’s instructions. qPCR was performed on the Bio-Rad S1000 with Bestar SYBR Green RT-PCR Master Mix (DBI Bioscience, Shanghai, China). The PCR conditions are consisted of denaturing at 95°C for 10 min, 40 cycles of denaturing at 95°C for 15 s, annealing and extension at 60°C for 1 min. PCR amplifications were performed in triplicate for each sample.

**Table 1:** The genes and primers used for qRT-PCR experiments.

Gene	Forward primer (5'-3')	Reverse primer (5'-3')
*IL1A*	GCTTCAAGGAGAATGTGGTGAT	CCAGGTCGTCATCGGTGAT
*IL8*	GTACAGAACTTCGATGCCAATG	GCCCACTCTCAATAACTCTCAG
*CCL8*	TGAGGTCCTTCCACTGATTATC	ACATAGCACACATCCACTTACA
*CXCL10*	TGGGCTTATTAGAGACCTTAGG	TGGCTGCTTCTGTATATTTGGA
*CXCL11*	ACAAGTGTAACTGTGACTACTG	GGAGACAGAGGTGCTTTCATA
*FAS*	CGTGGCTGGTATCAACTCTG	AGGAGGACAAGGCTGACAG
*IER3*	GGGAAGAAGTGCGTCGTTAA	CCCACAGAGCCCAATAAATACC
*FAIM*	TATTGATGCTGTCGGTGGTT	ACTCTAAAGTCCTCACTATCCA
*CAT*	AACTCAATGTTCTGACGGTAGG	GGTCAAAGTGAGCCATTTCATC
*KRT18*	GCTGACCGTGGAGTTGGAT	GCTCCTCTCGGTTCTTCTGA
*GAPDH*	GCGACACTCACTCTTCTACCT	TCTCTTCCTCTCGTGCTCCT

### Statistical analysis

All values were presented as mean ± SD. For comparison, the significance of differences between means was determined by Student’s *t-*test. A value *P<0*.*05* was regarded as statistically significant.

### Online data deposition

The RNA-seq data has been deposited in NCBI Gene Expression Omnibus(GEO) under accession code GSE95203.

## Results

### RNA-seq data summary and exploration of differentially expressed genes

The young small-tailed Han sheep were inoculated with ORFV, and we prepared the oral mucosa tissue samples (T0, T3, T7 and T15) on day 0, 3, 7 and 15 after ORFV infection, respectively. Totally, 8 cDNA libraries (T0-A, T0-B, T3-A, T3-B, T7-A, T7-B, T15-A and T15-B) were constructed for RNA-seq, which composed two biological replicates at each time point.

Through Illumina NextSeq 500, we generated over 0.18 billion pair-end reads, corresponding to an average of 22.5 million sequence reads per sample (S1 File). Using TopHat [25], 68% of all the reads were successfully mapped against the current sheep reference genome (ftp://ftp.ncbi.nlm.nih.gov/genomes/Ovis_aries) (S2 File).

**Fig 1:**
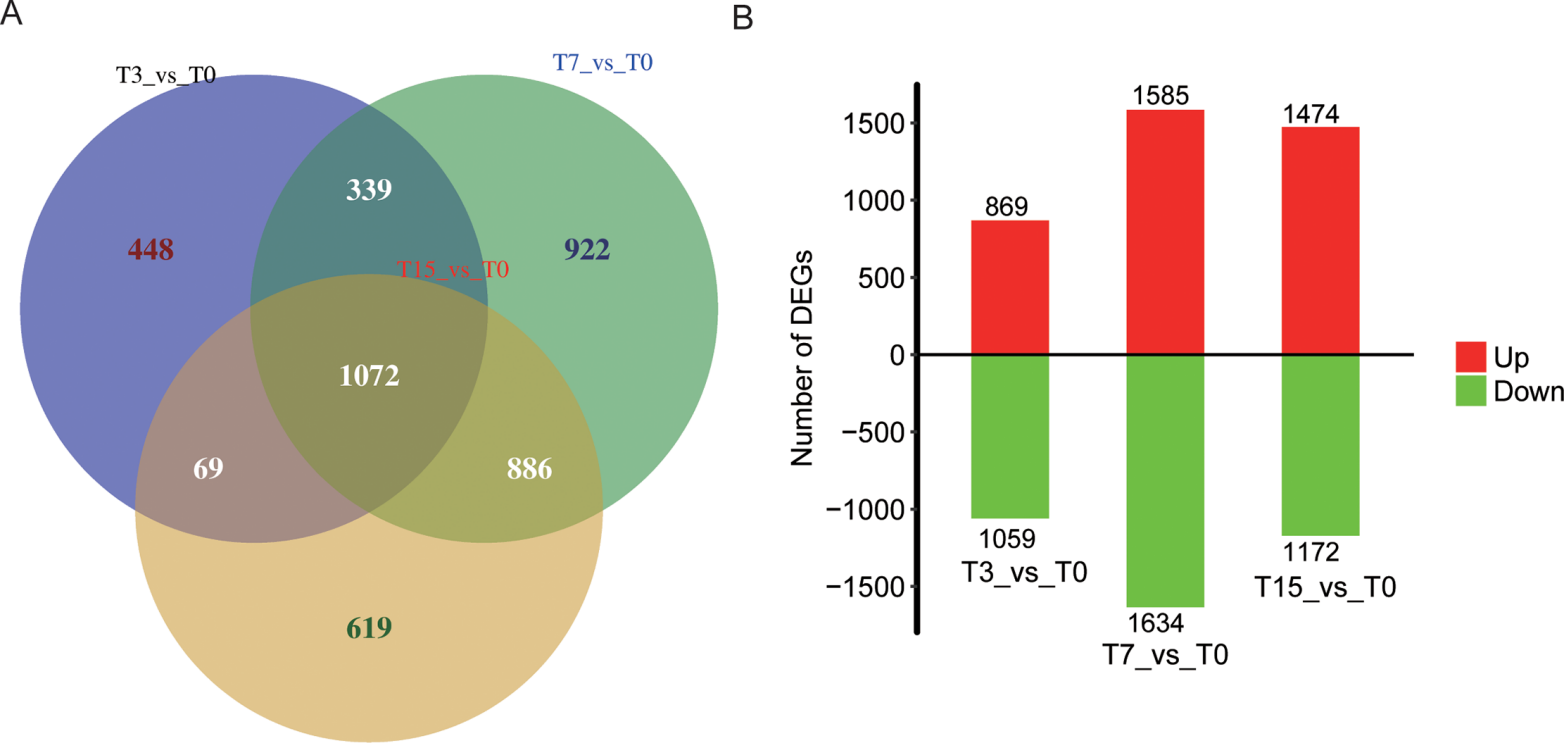
Exploration of differentially expressed genes (DEGs) (fold change ≥ 2.0 or ≤ 0.5, *P*≤0.01). (A) Venn diagrams of DEGs from T3 vs. T0, T7 vs.T0 and T15 vs.T0 comparisons. (B) The number of up and down-regulated DEGs in three comparisons.

Using software edgeR [22], a total of 1928, 3219 and 2646 differentially expressed genes (DEGs) (FC ≥ 2 or ≤ 0.5, *P* **≤** 0.01) were detected in the comparisons of T3 vs. T0, T7 vs. T0 and T15 vs. T0 respectively, with the highest or lowest FC being 2^12.7^ and 2^−10.47^ (Fig 1, S3 File). It indicated that ORFV infection could lead to comprehensive transcriptome changes of host cells from oral mucosa tissues. Moreover, there are 1072 common DEGs among three comparisons above, and many DEGs have immune function, implying that ORFV infection would mainly affect the immune system of the host skin.

### Immune response after ORFV infection

To identify the pathways in which the DEGs were mainly involved, Gene Ontology (GO) enrichment analysis was conducted. It showed that 43, 81 and 85 GO terms were identified in T3 vs. T0, T7 vs. T0 and T15 vs. T0, respectively (*P*<0.05) (S4 File).

**Fig 2:**
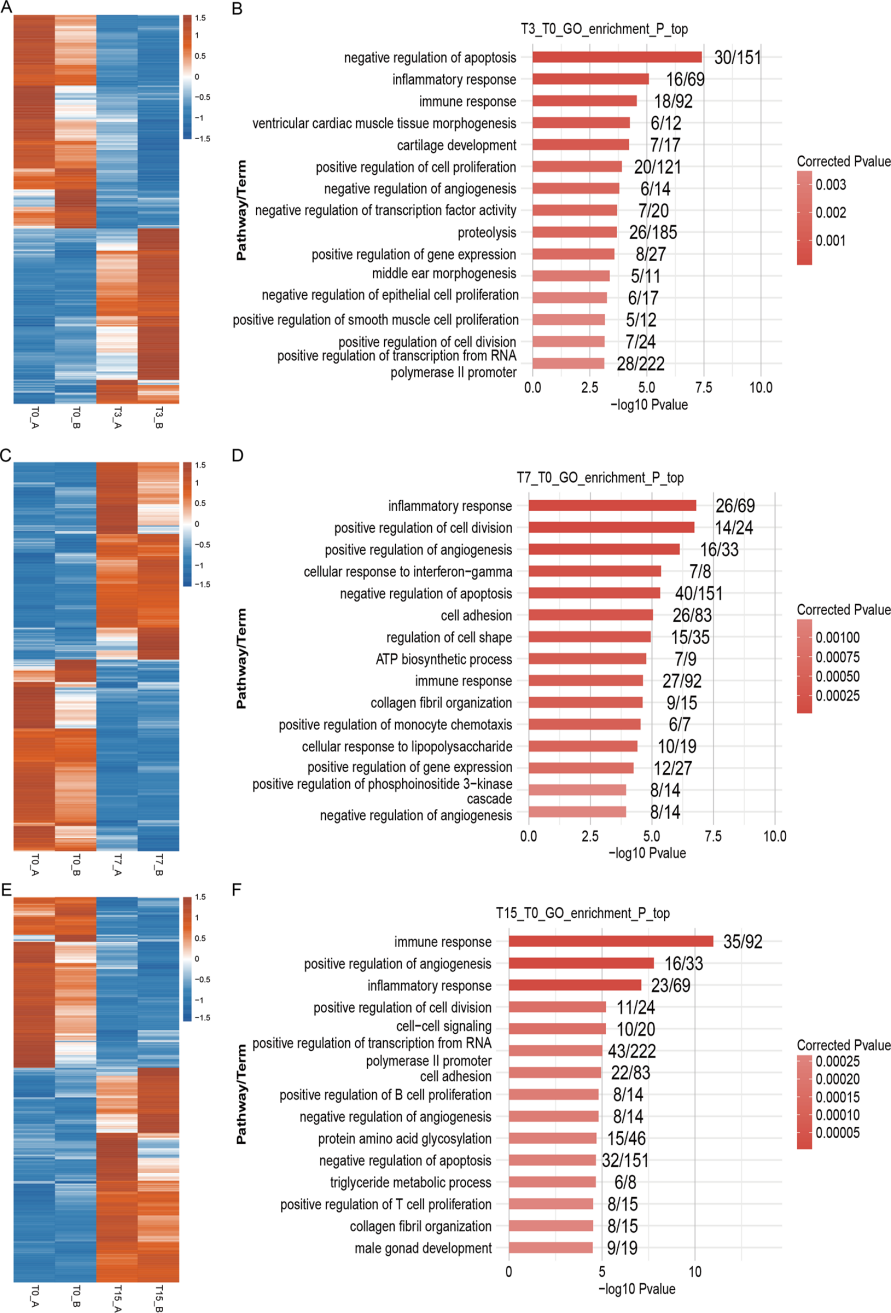
GO analysis of differentially expressed genes (DEGs) from T3 vs. T0, T7 vs. T0 and T15 vs. T0. (A), (C) & (E) The heatmaps for DEGs from *T3 vs*. *T0*, *T7 vs*.*T0* and *T15 vs*.*T0*, respectively. The heatmap was generated from hierarchical analysis of genes and samples. (B), (D) & (F) GO analyses of DEGs from *T3 vs*. *T0*, *T7 vs*.*T0* and *T15 vs*.*T0*, respectively. Only the top 15 terms are listed here.

**Fig 3:**
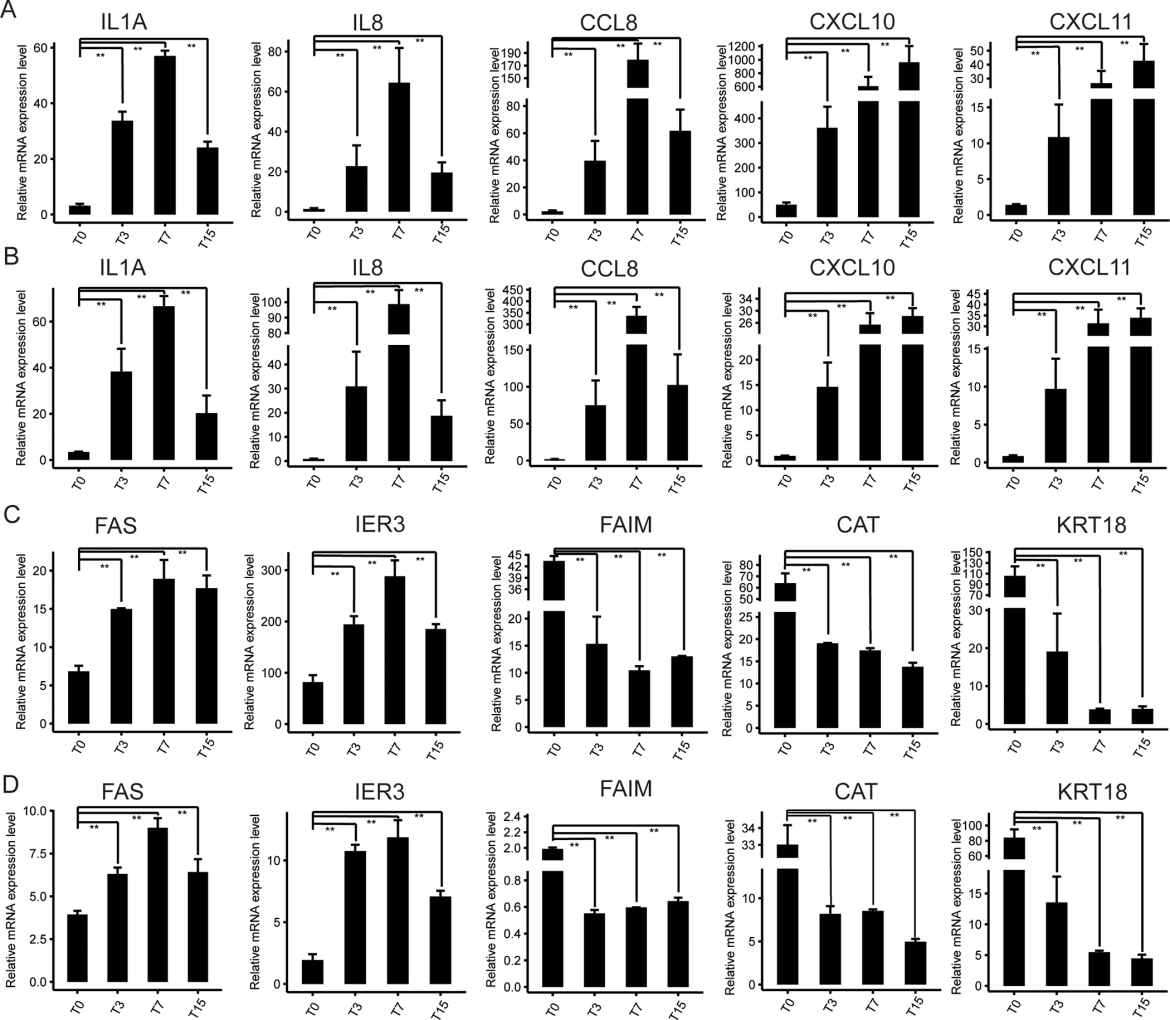
Quantitative real-time PCR (qRT-PCR) validation of some differentially expressed genes (DEGs) obtained from RNA-seq. (A) & (C) The mRNA expression levels of some DEGs associated with immune system and regulation of apoptosis were determined by high-throughput sequencing. RPKM (reads per kilobase per million mapped reads) was used to calculate the expression levels of genes. (B) & (D) The mRNA expression levels of some DEGs above were validated by qRT-PCR, normalized with the *GAPDH* gene. Data represent the mean values ±SD. *, *P*<0.05; **, *P*<0.01, calculated with student’s *t* test.

Multiple DEGs were enriched in “inflammatory response” (GO: 0006954), “immune response” (GO: 0006955), and “defense response to virus” (GO: 0051607) terms for all three comparisons (Fig 2B, 2D and 2F, S4 File), indicating that ORFV infection has evocated the immune system of the sheep. Previous studies revealed that ORFV infection had the capacity to cause immune response or immunosuppression of the host [26,27]. In order to explore how the ORFV infection affected the immune system, we analyzed these DEGs enriched in“inflammatory response” and “immune response” terms. It indicated that the expression level of multiple DEGs associated with immune system, including interleukin 1 Alpha (IL1A), interleukin 8 (IL8), C-C motif chemokine ligand 8 (CCL8), C-X-C motif chemokine ligand 10 (CXCL10) and C-X-C motif chemokine ligand 11 (CXCL11), were obviously up-regulated after ORFV infection (Fig 3A). IL1 is a pleiotropic cytokine involved in various immune responses, inflammatory processes, and hematopoiesis. IL8, CCL8, CXCL10 and CXCL11 are chemotactic proteins which can activate and regulate the inflammation process. Moreover, the results of qRT-PCR experiments were consistent with the results from RNA-seq (Fig 3B) with Pearson correlation coefficients (PCCs) higher than 0.9, suggesting that ORFV infection might provoke a vigorous immune response of the host cells from oral mucosa.

### Apoptosis of the host cell after ORFV infection

Apoptosis, a form of cell death, which is distinct from necrosis, is of great significance in processes such as homeostasis and the elimination of damaged cells, and could be triggered by cytokines and immune effector cells [28]. Previous studies indicated that apoptotic cell death forms part of the host defense against virus infection, and apoptosis of the host cell before the completion of the viral replication cycle may limit the number of progeny and the spread of infection [29].

**Fig 4:**
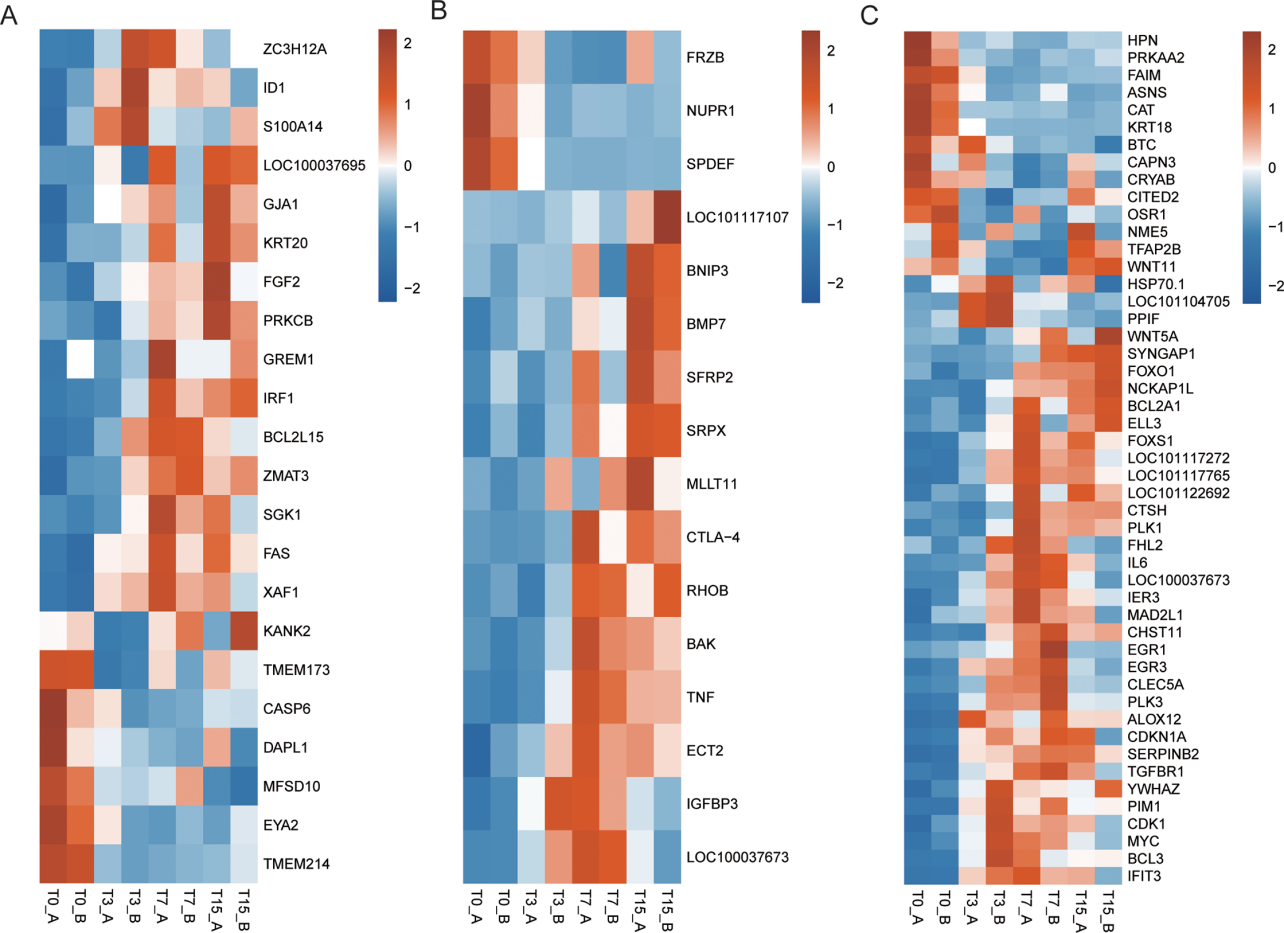
The differentially expressed genes (DEGs) were associated with regulation of apoptosis of host cells from oral mucosa tissue after the ORFV infection. (A) The heatmap of DEGs enriched in “apoptosis” (GO: 0006915) term; (B) The heatmap of DEGs enriched in “positive regulation of apoptosis” (GO: 0043065) term; (C) The heatmap of DEGs enriched in “negative regulation of apoptosis” (GO:0043066) term.

In the current study, it was uncertain whether ORFV could lead to apoptosis of host cells from oral mucosa tissues, but “apoptosis” (GO: 0006915) term could be found in T3 vs. T0, and T15 vs. T0 (S4 File). Although the “apoptosis” term could not be identified in the T7 vs. T0, many DEGs involved in apoptotic pathways could be found (Fig 4A, S3 File). Moreover, the expression levels of most DEGs enriched in “apoptosis” term were up-regulated after the ORFV infection, including *Fas* (Fas cell surface death receptor) gene (Fig 3C and 3D), which was an apoptosis biomarker in the physiological regulation of programmed cell death [30]. It suggested that host cells might defend the ORFV invasion through activating pro-apoptotic genes during the early stages of infection.

### Positive and negative regulation of apoptosis after ORFV infection

Upon virus invasion into the host, a fierce and complex battle occurs between the host cells and viruses. The host cells will defend virus proliferation through different ways including apoptosis; meanwhile, these viruses have developed a vast array of modulators that block apoptosis at different stages within the apoptotic pathways [14,15]. Interestingly, the ORFV125 encoded by ORFV, was a Bcl-2-like inhibitor of apoptosis [16], implying there might be some molecular mechanisms of regulating apoptosis.

From the results of GO analysis, the term of “positive regulation of apoptosis” (GO: 0043065) was only found in T15 vs. T0 (S4 File), but many DEGs belonging to “apoptosis” term could be found in T3 vs. T0 and T7 vs. T0 (S3 File and Fig 4B). The expression levels of most DEGs enriched in this term were up-regulated after ORFV infection, including *Bak* and *Tnf* genes which have been shown to induce apoptosis [31,32], suggesting the host cells might defend ORFV invasion through up-regulating some genes’ expression levels.

Moreover, the “negative regulation of apoptosis” (GO: 0043066) term could be found in T3 vs. T0, T7 vs. T0 and T15 vs. T0. Similarly, the expression levels of most DEGs enriched in this pathway were up-regulated after ORFV infection, including *IER3* (immediate early response 3) gene which has been reported to inhibit apoptosis [33] (Figs 3C, 3D and 4C), suggesting that ORFV might inhibit apoptosis of host cells by up-regulating the expression levels of some genes for its replication. Therefore, it indicated that serious interaction between mammalian host and ORFV generated at the early stages of infection.

### Network analysis of DEGs regulating apoptosis

**Fig 5:**
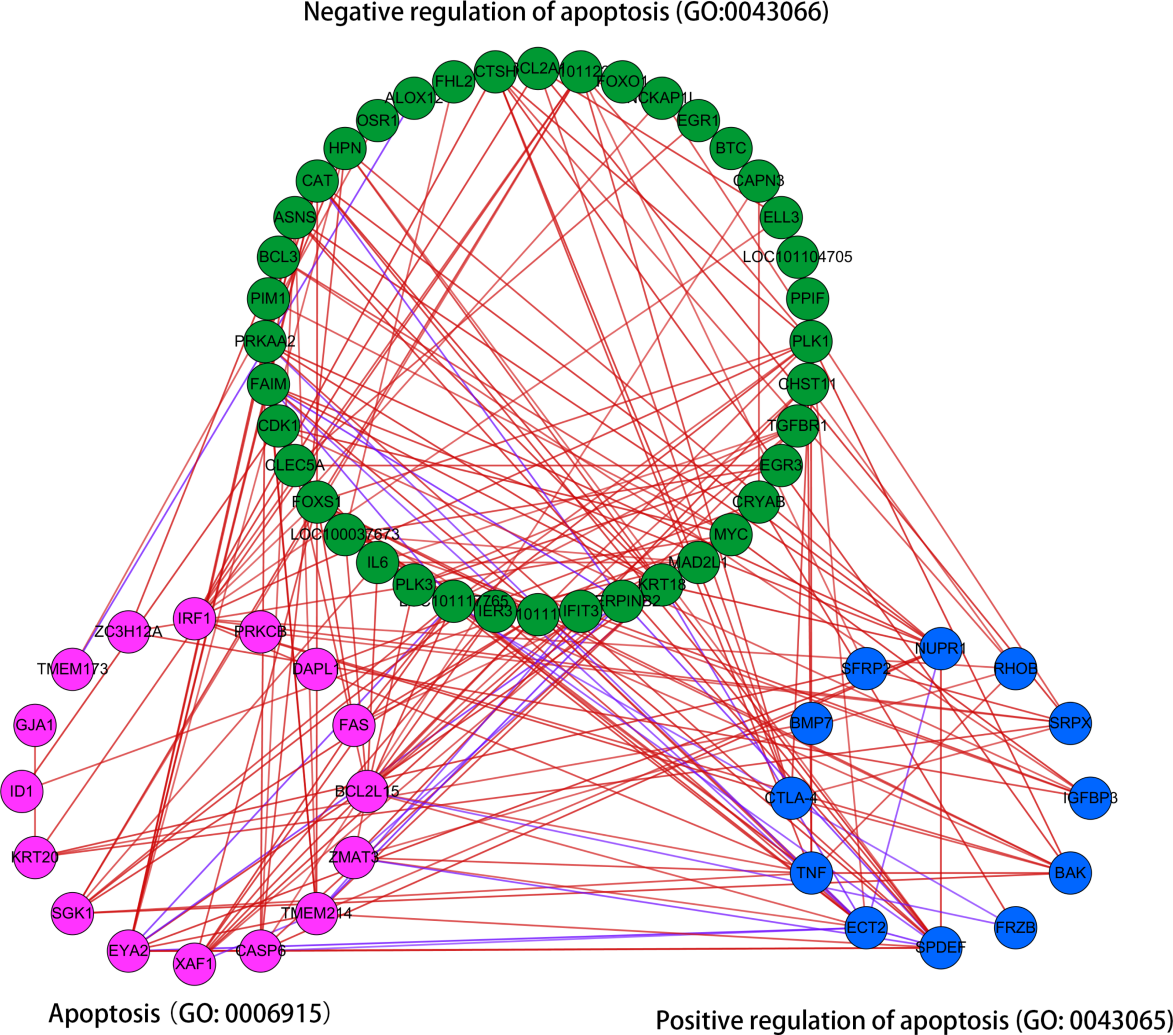
The co-expression analysis of differentially expressed genes (DEGs) regulating apoptosis. The network analysis under the Pearson correlation coefficients (PCCs) of these DEGs (PPC ≥ 0.9, P<0.01) enriched in “apoptosis” (GO: 0006915), “Positive regulation of apoptosis” (GO: 0043065) and “negative regulation of apoptosis” (GO:0043066) terms. The nodes represent DEGs. Red lines represent positive correlations, and blue lines signify negative correlation.

The results above revealed that the positive and negative regulation mechanisms of apoptosis could co-exist in the ORFV-infected cells. In order to explore the co-expression relationships of the DEGs, the network was constructed under the PCCs among these DEGs (PPC ≥ 0.90, *P*<0.01) which were enriched in “apoptosis”, “positive regulation of apoptosis” and “negative regulation of apoptosis” pathways (Fig 5). The number of analyzed genes was 79, with 68 out of them being well linked. There were 27 links between “positive regulation of apoptosis” and “apoptosis” pathways, while 60 links existed between “negative regulation of apoptosis” and “apoptosis” pathways, without significant difference (P>0.05).

The fabulous co-expression relationships among these DEGs indicated that positive and negative molecular mechanisms worked as a whole to regulate apoptosis, in order to reach a homeostasis of oral mucosa tissue after ORFV infection.

## Discussion

ORFV is a prototype species of *Parapoxvirus* in Poxviridae which includes *Bovine papular stomatitis virus* (BPSV), *Pseudocowpox* (PCPV) and *Parapoxviruses of red deer in New Zealand* (PVNZ) [6]. Generally, sheep and goats are natural hosts of ORFV, but infections have also been occasionally reported in other animals, even in human beings. Although ORFV mainly exists in sheep and goat with world-wide distribution, no evidence has shown the systemic spread of the virus yet [34], indicating that the host could provide protection against infection. ORFV usually infects the host through the injury and abrasions of the skin, and it replicates in regenerating keratinocytes. Under normal conditions, the lesions are benign, but more serious complications can take place when the natural hosts were infected again via bacteria or fungi. We may not take Orf as a serious disease because of its moderate and self-limiting clinical presentation, and recover completely with routine wound care and antibiotic agents. however, there might be a far-reaching economic impact, especially in some endemic areas, where the disease has spread seriously [5]. For preventing the outbreak of the disease, some ORFV attenuated live vaccines have been developed in the past few decades[35–37], and they indeed play an important role in blocking Orf in sheep and goats. However, all available vaccines do not have the capacity to induce persistent immunity in sheep and goats[38]. So far, there is no specific treatment for ORFV infections. Although many different strategies such as use of cidofovir, imiquimod, shave excision, curettage, cryotherapy, and electrocautery have all been reported to be successful, there is no supporting evidence from controlled clinical trials[39–44]. Therefore, Orf could not be underestimated, and it is necessary to explore the interaction mechanism between the host and ORFV.

Skin provides the essential protection from injury and infection for mammals. The cellular immune system of skin, as well as the associated lymphatic organs, have developed from constant exposure to microbial pathogens during the evolutionary process. Therefore, they can respond to such organisms fast and efficiently. Previous studies revealed that ORFV infection might provoke vigorous skin immune responses of the host to block virus replication, but ORFV could also produce some factors and interference with host immune mechanisms [34], to allow time for its replication. In the current study, analysis of RNA-Seq data from oral mucosa tissues of the host indicated that inflammation and immune response happened rapidly after ORFV infection (Fig 2). Mammalian interleukin 10 (IL10) is a cytokine suppressing inflammation and immune responses, and it revealed that ORFV could also produce a similar anti-inflammatory virokine (ORFV-IL-10) and knockout of this gene seriously attenuated the virus [45]. The presence of viral immuno-modulatory virulence factors was not investigated in the current study, but it was found that the expression level of IL-10 of the ORFV-infected cells was up-regulated after infection (S3 File), suggesting the ORFV could limit the effectiveness of host immunity through promoting the anti-inflammatory signals in the host cells.

Despite the presence of viral immuno-modulatory virulence factors in ORFV, strong inflammatory responses were observed the first few days (Day 3 and 7) of infection (Figs 2 and 3). Similarly, robust local inflammation was observed in variola virus and vaccinia virus infection[46,47]. And increasing evidence is suggesting that the uncontrolled inflammation is the major cause of significant pathology and lethality in poxvirus infection[48,49]. Therefore, in terms of contagious ecthyma treatment, rather than antivirals targeting the pathogen only, a combination therapy approach involving antivirals along with immunotherapy such as anti-inflammation drugs may produce a more favorable outcome with limiting local lesions and tissue damage. Apoptosis, a process of programmed cell death, really matters in forming an important host defense mechanism to limit virus infection. Infected cells can recognize virus particles at the cell entry, viral proteins and DNA/RNA during early viral replication, and in response, carry out the suicide program to block virus replication [14]. However, the viruses have developed various strategies to stop apoptosis [14,15]. In the current study, it revealed that complex molecular regulations of apoptosis function together in ORFV-infected cells at comprehensive transcriptome level through up or down-regulating the expression levels of genes involved in apoptotic pathways. For example, the expression levels of some DEGs enriched in “negative regulation of apoptosis” functional pathway were up-regulated after infection, but other DEGs’ expression levels were down-regulated (including *FAIM*, *CAT* and *KRT18*) (Figs 3C, 3D and 4C). It further suggested that both the positive and negative molecular mechanisms work integratedly to regulate apoptosis, in order to reach a homeostasis of host cells. It may explain why it takes a long period of time to recover from the primary ORFV infections and re-infections occur frequently.

## Supporting information

S1 File Data summary of RNA-Seq. (XLS)

S2 File Mapping of clean reads on the reference genome. (XLS)

S3 File Differentially expressed genes. (XLS)

S4 File GO term enrichment for the differentially expressed genes. (XLS)

## References

[1] Inoshima Y, Murakami K, Wu D, Sentsui H. Characterization of parapoxviruses circulating among wild Japanese serows (Capricornis crispus). Microbiology and immunology. 2002; 46(8): 583–7. 12363024 10.1111/j.1348-0421.2002.tb02738.x

[2] Spyrou V, Valiakos G. Orf virus infection in sheep or goats. Veterinary microbiology. 2015; 181(1–2): 178–82. doi: 10.1016/j.vetmic.2015.08.010 26315771 10.1016/j.vetmic.2015.08.010

[3] Kumar N, Wadhwa A, Chaubey KK, Singh SV, Gupta S, Sharma S, et al Isolation and phylogenetic analysis of an orf virus from sheep in Makhdoom, India. Virus Genes. 2014; 48(2): 312–9. doi: 10.1007/s11262-013-1025-9 10.1007/s11262-013-1025-9 24347045

[4] Haig DM, McInnes CJ, Thomson J, Wood A, Bunyan K, Mercer A. The orf virus OV20.0L gene product is involved in interferon resistance and inhibits an interferon-inducible, double-stranded RNA-dependent kinase. Immunology. 1998, 93(3): 335–40. 9640243 10.1046/j.1365-2567.1998.00438.x PMC1364081

[5] Haig DM. Orf virus infection and host immunity. Current opinion in infectious diseases. 2006; 19(2): 127–1. doi: 10.1097/01.qco.0000216622.75326.ef 16514336 10.1097/01.qco.0000216622.75326.ef

[6] Fleming SB, Wise LM, Mercer AA. () Molecular genetic analysis of orf virus: a poxvirus that has adapted to skin. Viruses. 2015; 7(3): 1505–39. doi: 10.3390/v7031505 25807056 10.3390/v7031505 PMC4379583

[7] Cyster JG.Chemokines, sphingosine-1-phosphate, and cell migration in secondary lymphoid organs. Annual review of immunology. 2005;23: 127–59. doi: 10.1146/annurev.immunol.23.021704.115628 15771568 10.1146/annurev.immunol.23.021704.115628

[8] Lateef Z, Baird MA, Wise LM, Young S, Mercer AA, Fleming SB. () The chemokine-binding protein encoded by the poxvirus orf virus inhibits recruitment of dendritic cells to sites of skin inflammation and migration to peripheral lymph nodes. Cellular microbiology. 2010; 12(5): 665–76. doi: 10.1111/j.1462-5822.2009.01425.x 20039877 10.1111/j.1462-5822.2009.01425.x

[9] Diel DG, Luo S, Delhon G, Peng Y, Flores EF, Rock DL. Orf virus ORFV121 encodes a novel inhibitor of NF-kappaB that contributes to virus virulence. Journal of virology. 2011; 85(5): 2037–49. doi: 10.1128/JVI.02236-10 21177808 10.1128/JVI.02236-10 PMC3067802

[10] Diel DG, Luo S, Delhon G, Peng Y, Flores EF, Rock DL. A nuclear inhibitor of NF-kappaB encoded by a poxvirus. Journal of virology. 2011; 85(1): 264–75. doi: 10.1128/JVI.01149-10 20980501 10.1128/JVI.01149-10 PMC3014193

[11] Diel DG, Delhon G, Luo S, Flores EF, Rock DL. A novel inhibitor of the NF-{kappa}B signaling pathway encoded by the parapoxvirus orf virus. Journal of virology. 2010; 84(8): 3962–73. doi: 10.1128/JVI.02291-09 20147406 10.1128/JVI.02291-09 PMC2849485

[12] Jin Z, El-Deiry WS. Overview of cell death signaling pathways. Cancer biology & therapy.2005; 4(2): 139–63. 15725726 10.4161/cbt.4.2.1508

[13] Lawen A. () Apoptosis-an introduction. Bioessays. 2003; 25(9): 888–96. doi: 10.1002/bies.10329 12938178 10.1002/bies.10329

[14] Everett H, McFadden G. Apoptosis: an innate immune response to virus infection. Trends in microbiology. 1999; 7(4): 160–5. 10217831 10.1016/s0966-842x(99)01487-0

[15] Everett H, McFadden G. Poxviruses and apoptosis: a time to die. Current opinion in microbiology. 2002; 5(4): 395–402. 12160859 10.1016/s1369-5274(02)00340-5

[16] Westphal D, Ledgerwood EC, Hibma MH, Fleming SB, Whelan EM, Mercer AA. A novel Bcl-2-like inhibitor of apoptosis is encoded by the parapoxvirus ORF virus.Journal of virology. 2007; 81(13): 7178–88. doi: 10.1128/JVI.00404-07 17475653 10.1128/JVI.00404-07 PMC1933275

[17] Westphal D, Ledgerwood EC, Tyndall JD, Hibma MH, Ueda N, Fleming SB, et al The orf virus inhibitor of apoptosis functions in a Bcl-2-like manner, binding and neutralizing a set of BH3-only proteins and active Bax. Apoptosis. 2009; 14(11): 1317–30. doi: 10.1007/s10495-009-0403-1 19779821 10.1007/s10495-009-0403-1

[18] Tian H, Chen Y, Wu J, Lin T, Liu X. Identification and function analysis of the host cell protein that interacted with Orf virus Bcl-2-like protein ORFV125. Research in veterinary science. 2016; 108: 93–7. doi: 10.1016/j.rvsc.2016.08.005 27663376 10.1016/j.rvsc.2016.08.005

[19] Devadas K, Biswas S, Haleyurgirisetty M, Wood O, Ragupathy V, Lee S, et al Analysis of Host Gene Expression Profile in HIV-1 and HIV-2 Infected T-Cells. PLoS One. 2016; 11: e0147421 doi: 10.1371/journal.pone.0147421 26821323 10.1371/journal.pone.0147421 PMC4731573

[20] Chen S, Wang A, Sun L, Liu F, Wang M, Jia R, et al Immune-Related Gene Expression Patterns in GPV- or H9N2-Infected Goose Spleens. International journal of molecular sciences. 2016; 17(12). 10.3390/ijms17121990 PMC5187790 27916934

[21] Huang Y, Li Y, Burt DW, Chen H, Zhang Y, Qian W, et al The duck genome and transcriptome provide insight into an avian influenza virus reservoir species. Nature genetics. 2013; 45(7): 776–83. doi: 10.1038/ng.2657 23749191 10.1038/ng.2657 PMC4003391

[22] Robinson MD, McCarthy DJ, Smyth GK. edgeR: a Bioconductor package for differential expression analysis of digital gene expression data. Bioinformatics. 2010; 26(1): 139–40. doi: 10.1093/bioinformatics/btp616 19910308 10.1093/bioinformatics/btp616 PMC2796818

[23] Huang DW, Sherman BT, Lempicki RA. Systematic and integrative analysis of large gene lists using DAVID bioinformatics resources. Nature protocols. 2009; 4(1): 44–57. doi: 10.1038/nprot.2008.211 19131956 10.1038/nprot.2008.211

[24] Shannon P, Markiel A, Ozier O, Baliga NS, Wang JT, Ramage D, et al Cytoscape: a software environment for integrated models of biomolecular interaction networks. Genome research. 2003;13(11): 2498–504. doi: 10.1101/gr.1239303 14597658 10.1101/gr.1239303 PMC403769

[25] Trapnell C, Pachter L, Salzberg SL. TopHat: discovering splice junctions with RNA-Seq. Bioinformatics. 2009; 25(9): 1105–11. doi: 10.1093/bioinformatics/btp120 19289445 10.1093/bioinformatics/btp120 PMC2672628

[26] Zaharia D, Kanitakis J, Pouteil-Noble C, Euvrard S. Rapidly growing orf in a renal transplant recipient: favourable outcome with reduction of immunosuppression and imiquimod. Transplant international. 2010; 23(10): e62–4. doi: 10.1111/j.1432-2277.2010.01147.x 20681978 10.1111/j.1432-2277.2010.01147.x

[27] Bennett JR, Lateef Z, Fleming SB, Mercer AA, Wise LM. Orf virus IL-10 reduces monocyte, dendritic cell and mast cell recruitment to inflamed skin. Virus research. 2016; 213: 230–7. doi: 10.1016/j.virusres.2015.12.015 26732486 10.1016/j.virusres.2015.12.015

[28] Wonderlich ER, Swan ZD, Bissel SJ, Hartman AL, Carney JP, O'Malley KJ, et al Widespread Virus Replication in Alveoli Drives Acute Respiratory Distress Syndrome in Aerosolized H5N1 Influenza Infection of Macaques. Journal of immunology. 2017; 98(4):1616–26. 10.4049/jimmunol.1601770 PMC5751439 28062701

[29] Cuff S, Ruby J. Evasion of apoptosis by DNA viruses. Immunology and cell biology. 1996; 74(6): 527–37. doi: 10.1038/icb.1996.86 8989591 10.1038/icb.1996.86

[30] Galani V, Tatsaki E, Bai M, Kitsoulis P, Lekka M, Nakos G, et al The role of apoptosis in the pathophysiology of Acute Respiratory Distress Syndrome (ARDS): an up-to-date cell-specific review. Pathology research and practice. 2010; 206(3): 145–50. 10.1016/j.prp.2009.12.002 20097014

[31] Klee M, Pallauf K, Alcala S, Fleischer A, Pimentel-Muinos FX. Mitochondrial apoptosis induced by BH3-only molecules in the exclusive presence of endoplasmic reticular Bak. EMBO journal. 2009; 28(12): 1757–68. doi: 10.1038/emboj.2009.90 19339988 10.1038/emboj.2009.90 PMC2699367

[32] Gozzelino R, Sole C, Llecha N, Segura MF, Moubarak RS, Iglesias-Guimarais V, et al BCL-XL regulates TNF-alpha-mediated cell death independently of NF-kappaB, FLIP and IAPs. Cell research. 2008; 18(10): 1020–36. doi: 10.1038/cr.2008.76 10.1038/cr.2008.76 18591962

[33] Hamidi T, Algul H, Cano CE, Sandi MJ, Molejon MI, Riemann M, et al Nuclear protein 1 promotes pancreatic cancer development and protects cells from stress by inhibiting apoptosis. Journal of clinical investigation. 2012; 122(6): 2092–103. doi: 10.1172/JCI60144 22565310 10.1172/JCI60144 PMC3366404

[34] Haig DM, McInnes CJ. Immunity and counter-immunity during infection with the parapoxvirus orf virus. Virus research. 2002; 88(1–2): 3–16. 12297324 10.1016/s0168-1702(02)00117-x

[35] Nettleton PF, Brebner J, Pow I, Gilray JA, Bell GD, Reid HW, et al Tissue culture-propagated orf virus vaccine protects lambs from orf virus challenge. Veterinary record. 1996; 138(8): 184–6. 8677620 10.1136/vr.138.8.184

[36] Pye D. Vaccination of sheep with cell culture grown orf virus. Australian veterinary journal. 1990; 67(5): 182–6. 2378601 10.1111/j.1751-0813.1990.tb07751.x

[37] Mayr A, Herlyn M, Mahnel H, Danco A, Zach A, Bostedt H, et al [Control of ecthyma contagiosum (pustular dermatitis) of sheep with a new parenteral cell culture live vaccine]. Zentralblatt fur veterinarmedizin, Reihe B. 1981; 28(7): 535–52. 7331595

[38] Zhao K, He W, Gao W, Lu H, Han T, Li J, et al Orf virus DNA vaccines expressing ORFV 011 and ORFV 059 chimeric protein enhances immunogenicity. Journal of virology. 2011; 8: 562. 10.1186/1743-422X-8-562 PMC3269396 22204310

[39] Al-Qattan MM. Orf infection of the hand. Journal of hand surgery-american volume. 2011; 36(11): 1855–8. 10.1016/j.jhsa.2011.08.019 21975099

[40] Kilic SS, Puel A, Casanova JL. Orf Infection in a Patient with Stat1 Gain-of-Function. Journal of clinical immunology. 2015; 35(1): 80–3. doi: 10.1007/s10875-014-0111-7 25367169 10.1007/s10875-014-0111-7

[41] Lederman ER, Green GM, DeGroot HE, Dahl P, Goldman E, Greer PW, et al Progressive ORF virus infection in a patient with lymphoma: successful treatment using imiquimod. Clinical infectious diseases.2007; 44(11): e100–3. doi: 10.1086/517509 17479930 10.1086/517509

[42] Bergqvist C, Kurban M, Abbas O. Orf virus infection. Reviews in medical virology. 2017; 27(4). 10.1002/rmv.1932 28480985

[43] Caravaglio JV, Khachemoune A. Orf Virus Infection in Humans: A Review With a Focus on Advances in Diagnosis and Treatment. Journal of drugs in dermatology. 2017; 16(7): 684–9. 28697220

[44] Demiraslan H, Dinc G, Doganay M. An Overview of Orf Virus Infection in Humans and Animals. Recent patents on Anti-Infective drug discovery. 2017; [Epub ahead of print]. 10.2174/1574891X12666170602080301 28571550

[45] Fleming SB, Anderson IE, Thomson J, Deane DL, McInnes CJ, McCaughan CA, et al Infection with recombinant orf viruses demonstrates that the viral interleukin-10 is a virulence factor. Journal of general virology. 2007; 88(Pt7): 1922–7. 17554023 10.1099/vir.0.82833-0

[46] Ahmed CM, Dabelic R, Waiboci LW, Jager LD, Heron LL, Johnson HM. SOCS-1 mimetics protect mice against lethal poxvirus infection: identification of a novel endogenous antiviral system. Journal of virology. 2009; 83(3): 1402–15. doi: 10.1128/JVI.01138-08 19019946 10.1128/JVI.01138-08 PMC2620917

[47] Stanford MM, McFadden G, Karupiah G, Chaudhri G. Immunopathogenesis of poxvirus infections: forecasting the impending storm. Immunology and cell biology. 2007; 85(2): 93–102. doi: 10.1038/sj.icb.7100033 17228320 10.1038/sj.icb.7100033

[48] Jahrling PB, Hensley LE, Martinez MJ, Leduc JW, Rubins KH, Relman DA, et al (2004) Exploring the potential of variola virus infection of cynomolgus macaques as a model for human smallpox. Proceedings of the National Academy of Sciences of the United States of America. 2004; 101(42): 15196–200. doi: 10.1073/pnas.0405954101 15477589 10.1073/pnas.0405954101 PMC523454

[49] Rubins KH, Hensley LE, Jahrling PB, Whitney AR, Geisbert TW, Huggins JW, et al (2004) The host response to smallpox: analysis of the gene expression program in peripheral blood cells in a nonhuman primate model. Proceedings of the National Academy of Sciences of the United States of America. 2004; 101(42): 15190–5. doi: 10.1073/pnas.0405759101 15477590 10.1073/pnas.0405759101 PMC523453

